# Prediction of Gene Expression Patterns With Generalized Linear Regression Model

**DOI:** 10.3389/fgene.2019.00120

**Published:** 2019-03-04

**Authors:** Shuai Liu, Mengye Lu, Hanshuang Li, Yongchun Zuo

**Affiliations:** ^1^College of Information Science and Engineering, Hunan Normal University, Changsha, China; ^2^College of Computer Science, Inner Mongolia University, Hohhot, China; ^3^College of Life Sciences, Inner Mongolia University, Hohhot, China; ^4^The State Key Laboratory of Reproductive Regulation and Breeding of Grassland Livestock, Inner Mongolia University, Hohhot, China

**Keywords:** cell reprogramming, Oct4, transcription factor binding site (TFBS), combination intensity, generalized linear regression model, gene expression pattern, prediction

## Abstract

Cell reprogramming has played important roles in medical science, such as tissue repair, organ reconstruction, disease treatment, new drug development, and new species breeding. Oct4, a core pluripotency factor, has especially played a key role in somatic cell reprogramming through transcriptional control and affects the expression level of genes by its combination intensity. However, the quantitative relationship between Oct4 combination intensity and target gene expression is still not clear. Therefore, firstly, a generalized linear regression method was constructed to predict gene expression values in promoter regions affected by Oct4 combination intensity. Training data, including Oct4 combination intensity and target gene expression, were from promoter regions of genes with different cell development stages. Additionally, the quantitative relationship between gene expression and Oct4 combination intensity was analyzed with the proposed model. Then, the quantitative relationship between gene expression and Oct4 combination intensity at each stage of cell development was classified into high and low levels. Experimental analysis showed that the combination height of Oct4-inhibited gene expression decremented by a temporal exponential value, whereas the combination width of Oct4-promoted gene expression incremented by a temporal logarithmic value. Experimental results showed that the proposed method can achieve goodness of fit with high confidence.

## Introduction

Somatic cells can be reverted to a pluripotent stem cell by cell reprogramming. Cell reprogramming has been significant in many domains of biological and medical science, including tissue repair, organ reconstruction, disease pathogenesis, and new drug development (Wernig et al., 2007; Park et al., 2008). Earlier, the nuclear transfer method was the main method to cultivate new individuals. However, this method was very controversial in terms of ethics (Gurdon, 1958; Campbell et al., 1996; McCreath et al., 2000; Polejaeva et al., 2000). Recently, study of cells induced to reprogram through specific transcription factors became a hotspot. This method solved the problem of immune rejection of allogeneic cells. In this way, the patient-specific stem cells were obtained without ethical controversy (Lv et al., 2018; Poli et al., 2018; Stadhouders et al., 2018).

As an important regulatory element, transcription factor (TF) was involved in the regulation of transcription initiation, and binding sites of TFs in promoter regions affected gene expression (Duren et al., 2017). Oct4, a core transcription factor, played an important regulatory role in stem cell self-renewal and pluripotency maintenance. It controlled the development and differentiation of early embryos and was highly expressed in a variety of stem cells, including germ cells, embryonic stem cells (ESCs), embryonic germ cells (EGCs), and embryonic tumor cells. In an experiment of mice, Oct4 was observed to play a central role in the cellular pluripotency regulatory network, which reprogramed somatic cells into induced pluripotent stem cells (iPSCs) by expressing transcription factors Oct4, Sox2, Klf4, and c-Myc ectopically (Chen et al., 2016). Another study showed that pluripotent stem cells can be obtained by adding Oct3/4, Sox2, c-Myc, and Klf4 to the fiber cells of mice (Boyer et al., 2005). Regulation of these transcription factors on target genes was achieved mainly through the interaction of feedforward systems, self-regulatory networks and other signaling pathways (Boyer et al., 2005).

Oct4-binding sites in promoter regions were closely related to gene expression (Chen et al., 2016). However, the relationship between Oct4 combination intensity in promoter regions and gene expression remained unclear. Therefore, in this paper, a generalized linear regression model was proposed to analyze the relationship between gene expression and Oct4 combination intensity in promoter regions.

The rest of paper was organized as follows. section Related Work introduces related work on cell reprogramming and gene expression; section Materials and Methods provides materials and methods, including source of data, the proposed generalized linear regression model and evaluation criteria of model performance; section Results and Analysis contains detailed experimental results and analysis, including the solution result and performance analysis of our proposed model, analysis of factors affecting gene expression on every stage of cell development, and applications of our proposed model in gene classification; and section Conclusion summarizes the contents of this paper.

## Related Work

Previous studies reported mechanisms and methods of cell reprogramming. Earlier, Gurdon et al. applied the nuclear transfer method to cell reprogramming of *Xenopus laevis* (Gurdon, 1958). Campbell, McCreath, and Polejaeva cultivated cloning animals using nuclear transfer technology (Campbell et al., 1996; McCreath et al., 2000; Polejaeva et al., 2000). Håkelien and Hochedlinger analyzed a cell recombination mechanism based on nuclear fusion and nuclear transfer technology (Håkelien et al., 2002; Hochedlinger and Jaenisch, 2002). Later, Stadtfeld and Zardo analyzed the effects of specific transcription factors and epigenetic plasticity of chromatin on cell reprogramming (Stadtfeld et al., 2008; Zardo et al., 2008). Studies by Hanna and Li showed that overexpression of transcription factor Oct4 had an effect on cell reprogramming (Hanna et al., 2009; Li et al., 2009). Doege et al. elaborated the effects of the interaction of Oct4, Sox2, Klf4, and c-Myc on cell reprogramming in the early stages of cell reprogramming (Doege et al., 2012). Apostolou and Chen found that the dynamic mechanisms of chromatin change and DNA methylation had important effects on cell reprogramming (Apostolou and Hochedlinger, 2013; Chen et al., 2013). Koqa et al. analyzed the role of transcription factor Foxd1 in cell reprogramming (Koga et al., 2014). Recently, Poli and Stadhouders elaborated the roles of specific transcription factors used as inducing factors in cell reprogramming (Poli et al., 2018; Stadhouders et al., 2018).

The process of cell reprogramming was closely related to the regulation of gene expression. Moreover, regulation of gene expression is the molecular basis of many life activities, including cell differentiation, morphogenesis, and ontogeny (Chen et al., 2016). Earlier, Chen and Rimsky analyzed regulation effects of *cis-* and *trans-*regulatory elements on gene expression (Rimsky et al., 1989; Chen et al., 1990). Later, Ueda et al. analyzed effects of diurnal variation of transcription factors on gene expression (Ueda et al., 2002). Patricia et al. analyzed effects of the interaction of *cis-* and *trans-*regulatory elements on gene expression (Wittkopp et al., 2004). Sullivan CS et al. studied the regulation effect of microRNAs encoded by SV40 on gene expression (Sullivan et al., 2005). Jeffery et al. found factors related to gene expression using gene expression data and binding sites of transcription factor (Jeffery et al., 2007). Han et al. found that certain types of genomic organization by SATB1 had an effect on gene expression (Han et al., 2008). Afterward, Costa et al. predicted gene expression in T cell differentiation by using histone modification and binding affinity of transcription factor via a linear mixed model (Costa et al., 2011). Maienscheincline et al. searched for target genes regulated by transcription factors based on some information, including binding sites of transcription factors and target genes (Maienschein-Cline et al., 2012). MT and Holoch analyzed the effects of specific transcription factors and the regulation effect of RNA on gene expression, respectively (Lee et al., 2013; Holoch and Moazed, 2015). Recently, Engreitz and Singh clarified effects of lncRNA promoter, transcription factor, variable splicing, and histone modification on gene expression, respectively (Engreitz et al., 2016; Singh et al., 2016). Thomou and Wu analyzed effects of miRNAs and histone modifications on gene expression (Thomou et al., 2017; Wu et al., 2017). Additionally, Duren et al. predicted gene expression based on chromatin accessibility data, *cis-*acting and *trans*-acting element data by logistic regression models (Duren et al., 2017). Neumann and Stadhouders analyzed effects of LncRNA and the dynamic interaction of transcription factors with expression of target genes (Neumann et al., 2018; Stadhouders et al., 2018).

Many methods were proposed for deciphering regulation mechanisms of *cis*-regulatory and *trans*-regulatory elements based on gene expression. Studies showed that gene expression was closely related to Oct4 combination intensity in promoter regions (Machado et al., 2011; Machado, 2017; Yan et al., 2017; Antão et al., 2018). However, the quantitative relationship between gene expression and Oct4 combination intensity was not considered. Therefore, firstly, a generalized linear regression model was proposed for quantifying the relationship of gene expression and Oct4 combination intensity based on eight gene datapoints. Then, testing data were applied to test the generalization ability of the model. On the one hand, experiments of 27 genes, as well as all genes, from GEO were applied to analyze the quantitative relationship between Oct4 combination intensity and target gene expression at each stage of cell development by our proposed model. On the other hand, 27 genes were divided into positive and negative samples by our proposed method.

## Materials and Methods

### Datasets

Experimental data came from mouse transcriptome data and ChIP-seq data, which were downloaded from GEO database with accession numbers GSE67462 and GSE67520, respectively. In this paper, gene promoter regions were defined as −1.5 kb to +0.5 kb of gene transcription start sites (TSSs). For quantifying the relationship between gene expression and Oct4 combination intensity, while testing the generalization ability of the proposed model, experimental data were divided into training data and test data.

Training data were related to genes Btbd8, Cnbp, Cyb5r3, Dars2, Eef1a1, Hist1h2bf, Ptrh2, Zfp143, which were extracted based on the following steps.

Step 1. All dynamic Oct4 combination intensity and gene expression data related to genes Btbd8, Cnbp, Cyb5r3, Dars2, Eef1a1, Hist1h2bf, Ptrh2, Zfp143 were extracted from transcriptome and ChIP-seq data (Chen et al., 2016). Oct4 combination intensities were expressed as a series of peaks that contained three characteristics, including height, distance and width, which were defined as the value of the highest point corresponding to the midpoint of the peak (height); distance between the midpoint of the peak and transcription start site (distance); and difference between the right and left boundaries of the peak (width).

Step 2. Transcriptome and ChIP-seq data of the above genes from Day 0, Day 1, Day 3, Day 5, Day 7, Day 11, Day 15, and Day 18 were selected for studying the relation between time and gene expression (Chen et al., 2016).

Step 3. Promoter regions with the strongest signal were extracted to avoid the influence of redundant data.

Testing data were composed of two parts, including data of 27 genes and all genes. Firstly, 27 genes and all genes were applied to analyze quantitative relationship between Oct4 combination intensity and target gene expression at each stage of cell development by our proposed model. Then, 27 genes were divided into high and low expression groups to classify.

In detail, 27 genes were obtained by searching for those data that appeared in all eight different cell development stages from GEO. These genes were Alyref2, Atn1, Btbd8, Btg2, Caprin1, Cnbp, Ctgf, Cyb5r3, Dars2, Ddx5, Eef1a1, Fosb, Hes1, Hist1h2bb, Hist1h2bf, Hist1h2bp, Hnrnpa2b1, Kmt2e, Lonp1, Nfe2l2, Pecr, Phldb2, Ptrh2, Setd5, Trappc6b, Tti2, and Zfp143. In the bi-classification experiment, expression values of 27 genes were sorted by descending order. The top 30% of the sorted data were defined as the high expression group, and the lowest 30% were defined as the low expression group. The value of the minimum high expression was the threshold for classification.

The numbers of all genes at each stage of cell development are shown in Table 1.

**Table 1:** Number of genes at each cell development stage.

**Cell development stage**	**Number of genes at each** **stage**
Day 0	86
Day 1	4,062
Day 3	4,577
Day 5	4,101
Day 7	6,261
Day 11	7,984
Day 15	8,181
Day 18	6,485

### Generalized Linear Regression Model

**Figure 1:**
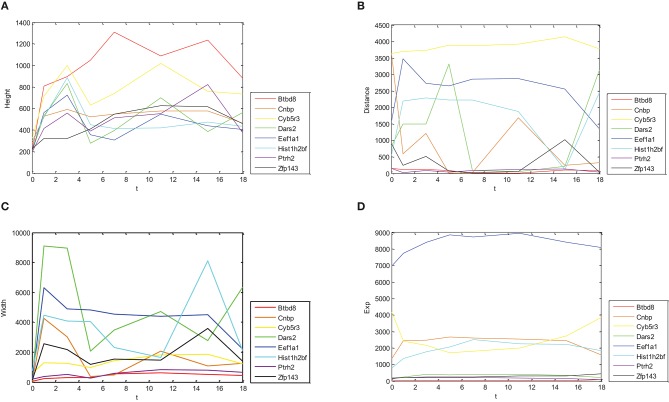
Dynamic change trends for expressions of different genes and their combination intensity. Dynamic change trends of height, distance, width and expression are shown in **(A–D)**, respectively. X axis represents time with 0, 1, 3, 5, 7, 11, 15, and 18 day(s). Y axis represents corresponding values of eight genes.

In Figure 1, relations between height, distance, width, gene expression of Oct4 combination intensity, and time were provided, respectively.

Figure 1 shows different change trends with time of Oct4 combination intensity in promoter regions and gene expression in the eight proposed genes. Figure 1A illustrates in detail that change trends of height with time were nearly identical in these genes. Similarly, Figure 1C demonstrates that change trends of width with time in these genes were also nearly identical. Figures 1B,D show that change trends of distance and gene expression with time were disorganized.

For quantifying the relationship between gene expressions and Oct4 combination intensity, correlations between height, distance, width, time, and gene expressions were analyzed by using their correlation coefficients, which is defined as Equation (1) with two random variables, *X* and *Y*.

(1) $$\begin{array}{r} \text{r}\left(X,Y\right)=\frac{cov\left(X,Y\right)}{\sqrt{var\left(X\right)\;var\left(Y\right)}} \end{array}$$

In Equation (1), *r*(*X, Y*) represents the correlation coefficient between *X* and *Y*, *cov*(*X, Y*) represents covariance between *X* and *Y*, *var*(*X*), and *var*(*Y*) represent variance of *X* and *Y*, respectively.

**Figure 2:**
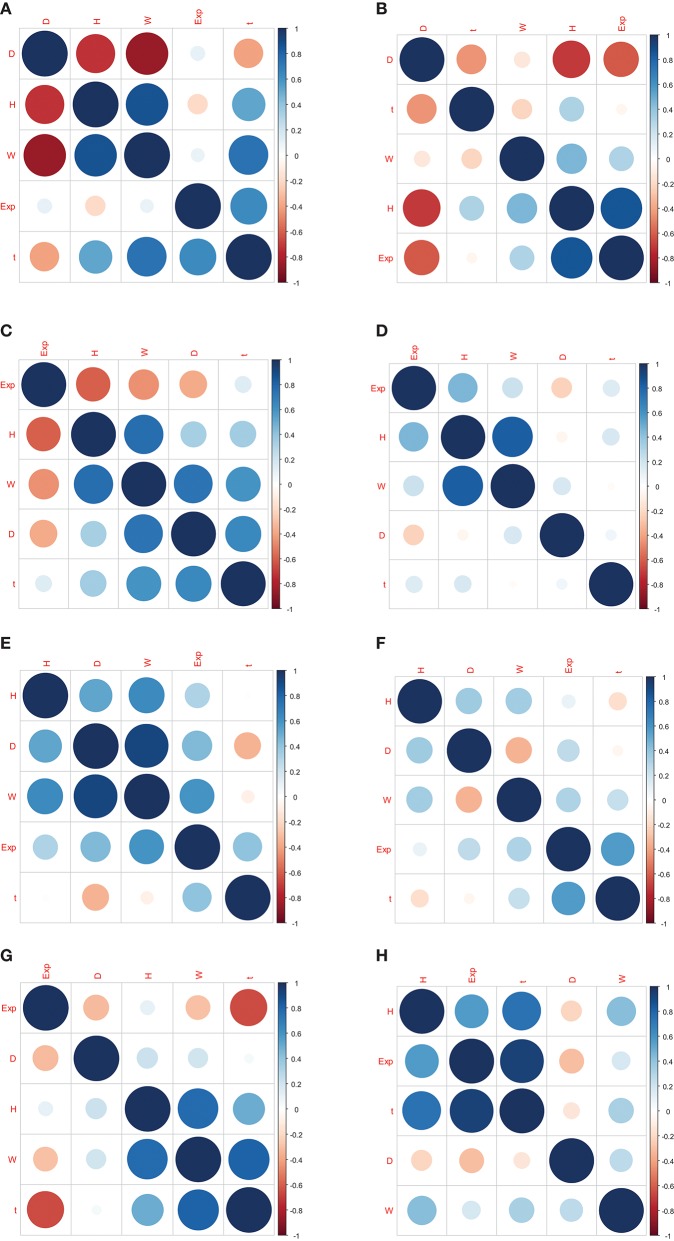
Correlation coefficients for Oct4 combination intensity and the gene expression, height, distance, width, and time of each gene. **(A–H)** represents the correlation coefficients in genes Btbd8, Cnbp, Cyb5r3, Dars2, Eef1a1, Hist1h2bf, Ptrh2 , Zfp143, respectively.

The correlation coefficients between gene expression and Oct4 combination intensity are shown in Table 2. In addition, correlation coefficients for Oct4 combination intensity and the gene expression, height, distance, width, and time of each gene are provided in Figure 2.

**Table 2:** Correlation coefficients between gene expression and Oct4 combination intensity for selected genes.

**Gene name**	**A11**	**A12**	**A12**	**A14**
Btbd8	−0.1983129	0.1048461	0.0906912	**0.6349461**
Cnbp	**0.85966104**	**−0.61927824**	0.30350817	−0.05763255
Cyb5r3	**−0.5934541**	−0.3768864	−0.4585991	0.1329110
Dars2	**0.4509573**	−0.2217020	0.2176128	0.1436256
Eef1a1	0.3075134	0.4400668	**0.5909714**	0.4070609
Hist1h2bf	0.09321187	0.25428558	0.30178739	**0.56638550**
Ptrh2	0.1068043	−0.3149051	−0.2933016	**−0.6536589**
Zfp143	**0.5607069**	−0.3091457	0.1798487	**0.9387029**

*A11, A12, A13, and A14 refer to correlation coefficients between gene expression and height, distance, width, and time, respectively. Bold text represents absolute values of correlation coefficients that are > 0.5*.

Table 2 and Figure 2 indicate that the correlation coefficients for gene expression and time were the largest. Correlation coefficients for time and other variables were also strong. However, goodness of fit was low when the predicted model was constructed using height, distance, and width as explanatory variables, and gene expression as explained variable. Due to the strong relationship between time and Oct4 combination intensity, several time-dependent derived combination variables were used as explanatory variables of the proposed model.

Firstly, new derived combination variables were obtained by multiplication operations between height, distance, width and a function of time *t*, including *e*^*t*^, log_10_(*t* + 1) and *t*^*k*^ (*k* = 1,2,3). In this way, a set V = {*H* × *t*, *H* × *t*^2^, *H* × *t*^3^, *H* × *e*^*t*^, *H* × 0.5^*t*^, *H* × log_10_(*t* + 1), *D* × *t*, *D* × *t*^2^, *D* × *t*^3^, *D* × *e*^*t*^, *D* × 0.5^*t*^, *D* × log_10_(*t* + 1), *W* × *t*, *W* × *t*^2^, *W* × *t*^3^, *W* × *e*^*t*^, *W* × 0.5^*t*^, *W* × log_10_(*t* + 1)} was constructed as the set of explanation variables, where H denotes height, D denotes distance and W denotes width. Then, stepwise regression method was used to determine explanatory parameters of the proposed regression model. Finally, six explanatory variables were selected from V, including *H* × *e*^*t*^, *D* × *t*, *D* × *t*^2^, *D* × *t*^3^, *D* × 0.5^*t*^ and *W* × log_10_(*t* + 1).

Therefore, a generalized linear regression model was constructed by using selected explanatory variables, in which gene expression was the explained variable. In this paper, four generalized linear regression models, Models 1–4, were constructed by Equations (2–5).

(2) $$\begin{array}{l} \text{Model 1}\;:\;\text{Exp}=\beta_{1}\times H\times e^{t}+\beta_{2}\times D\times t\\ \;+\beta_{3}\times W\times \log_{10}\left(t+1\right)+\varepsilon \end{array}$$

(3) $$\begin{array}{l} \begin{array}{c} \text{Model}\;2\;:\;\text{Exp}=\beta_{1}\times H\times e^{t}+\beta_{2}\times D\times t^{2} \end{array}\\ \;\begin{array}{c} +\beta_{3}\times W\times \log_{10}(t+1)+\varepsilon \end{array} \end{array}$$

(4) $$\begin{array}{l} \begin{array}{c} \text{Model}\;3\;:\;\text{Exp}=\beta_{1}\times H\times e^{t}+\beta_{2}\times D\times t^{3} \end{array}\\ \;\begin{array}{c} +\beta_{3}\times W\times \log_{10}(t+1)+\varepsilon \end{array} \end{array}$$

(5) $$\begin{array}{l} \begin{array}{l} \text{Model }4\;:\;\text{Exp}=\beta_{1}\times H\times e^{t}+\beta_{2}\times D\times 0.5^{t}\\ \;+\beta_{3}\times W\times \log_{10}(t+1)+\varepsilon \end{array} \end{array}$$

In Equations (2–5), Exp represents the value of gene expression; β_1_, β_2_, β_3_ are regression coefficients, which are calculated by the Least Squares Method (LSM), and LSM is defined as the sum of squares of differences between predicted value and true value; a random disturbance ε is a normal distribution that was applied to represent other factors affecting gene expression except height, distance and width.

*H* × *e*^*t*^ and *W* × log_10_(*t* + 1) were selected in the final model because they were common items in Equations (2–5). Therefore, a general model of gene expression patterns was obtained by Equation (6), and the correctness of the model will be verified in section Analysis of Factors Affecting Gene Expression at Every Stage of Cell Development.

(6) $$\begin{array}{l} \begin{array}{c} \text{Exp}=\beta_{1}\times H\times e^{t}+\beta_{2}\times D\times f(t) \end{array}\\ \;\begin{array}{c} +\beta_{3}\times W\times \log_{10}(t+1)+\varepsilon \end{array} \end{array}$$

In Equation (6), *f*(*t*) represents a function of time *t*, which was selected from {*t*, *t*^2^, *t*^3^, 0.5^*t*^}; β_1_, β_2_, and β_3_ are regression coefficients calculated by LSM.

### Evaluation Criteria of Model Performance

*F*-test, *t*-test, and goodness of fit ${\bar{R}}^{2}$ were used to evaluate the performance of linear regression model (Huang and Pan, 2003; Zhou et al., 2003; Xu et al., 2008; Wang and Lee, 2010; Wang et al., 2012). More precisely, *F*-test was used to test significance of the entire regression model and *t*-test was used to test significance of regression coefficients in the model. Goodness of fit ${\bar{R}}^{2}$ was used to measure the approximation degree between fitted curve and original data. Meanwhile, ${\bar{R}}^{2}$, a generation from original coefficient of determination *R*^2^, was an adjusted coefficient of determination. It was eliminated the influence of coefficient of determination generated by number of explanatory variables. In this paper, *F*-test statistic, *t*-test statistic, adjusted coefficient of determination ${\bar{R}}^{2}$, original coefficient of determination *R*^2^, total sum of squares (TSS), explained sum of squares (ESS), and residuals sum of squares (RSS) are defined as Equations (7–13) (Huang and Pan, 2003; Zhou et al., 2003; Xu et al., 2008; Wang and Lee, 2010; Wang et al., 2012).

(7) $$\begin{array}{r} F=\frac{ESS/k}{TSS/\left(n-k-1\right)}\tilde{\;}F\left(k,n-k-1\right) \end{array}$$

(8) $$\begin{array}{r} t=\frac{\hat{\beta_{j}}}{se\left(\hat{\beta_{j}}\right)}\tilde{\;}t\left(n-k-1\right) \end{array}$$

(9) $$\begin{array}{r} {\bar{R}}^{2}=1-\left(1-R^{2}\right)\frac{n-1}{n-k-1} \end{array}$$

(10) $$\begin{array}{r} R^{2}=1-\frac{RSS}{TSS}=1-\frac{\sum {\left(Y_{i}-{\hat{Y}}_{i}\right)}^{2}}{\sum {\left(Y_{i}-\overset{-}{Y}\right)}^{2}} \end{array}$$

(11) $$\begin{array}{r} \text{TSS}=\sum {y_{i}}^{2}={\left(Y_{i}-\overset{-}{Y}\right)}^{2} \end{array}$$

(12) $$\begin{array}{r} \text{ESS}=\sum {{\hat{y}}_{i}}^{2}={\left({\hat{Y}}_{i}-\overset{-}{Y}\right)}^{2} \end{array}$$

(13) $$\begin{array}{r} \text{RSS}=\sum {e_{i}}^{2}={\left(Y_{i}-{\hat{Y}}_{i}\right)}^{2} \end{array}$$

In Equations (7–13), *k* is the number of variables; *n* is the number of samples; ${\hat{\beta }}_{i}$ and $\text{se}\left({\hat{\beta }}_{i}\right)$ are estimated value and standard deviation of estimated value of regression coefficient; and *Y*_*i*_, Ŷ_*i*_, Ȳ represent true, estimated and mean values of explained variable.

Accuracy (Acc), Sensitivity (*S*_*n*_), specificity (*S*_*p*_), and Mathew correlation coefficient (Mcc) were used to measure the performance of the classification model (Xu et al., 2013; Guo et al., 2014; Awazu, 2016). Which were defined as Equations (14–17).

(14) $$\begin{array}{r} S_{n}=\frac{TP}{TP+FN} \end{array}$$

(15) $$\begin{array}{r} S_{p}=\frac{TN}{TN+FP} \end{array}$$

(16) $$\begin{array}{r} \text{Acc}=\frac{TP+TN}{TP+FN+TN+FP} \end{array}$$

(17) $$\begin{array}{l} \text{Mcc}=\frac{TP\times TN-FP\times FN}{\sqrt{\left(TP+FN\right)\times \left(TP+FP\right)\times \left(TN+FN\right)\times \left(TN+FP\right)}} \end{array}$$

In Equations (14–17), *TP* represents the number of positive samples that are correctly predicted as positive samples; *TN* represents the number of negative samples that are correctly predicted as negative samples; *FP* represents the number of negative samples that are incorrectly predicted as positive samples; and *FN* represents the number of positive samples that are incorrectly predicted as negative samples (Zhang et al., 2014, 2018; Wang et al., 2017, 2018a,b).

## Results and Analysis

### Solution Result of Our Proposed Model

Gene expression patterns of the eight selected genes were analyzed by using Models 1–4. More specifically, Model 1 was applied to describe the expression pattern of gene Zfp143, Model 2 was applied to describe the expression pattern of gene Hist1h2bf; Model 3 was applied to describe the expression patterns of genes Dars2 and Eef1a1, and Model4 was applied to describe the expression patterns of genes Btbd8, Cnbp, Cyb5r3, and Ptrh2. Both Model 2 and Model 3 were used to express the expression pattern of gene Eef1a1. Parameter values of the models are shown in Table 3. Parameter values of Model 2 and Model 3 for gene Eef1a1 were shown in Table 4.

**Table 3:** Parameter values of model for eight genes.

**Gene name**	**Regression coefficient**		
	**β_1_**	**β_2_**	**β_3_**
Btbd8	6.690e-10	**8.420e-02**	1.053e-02
Cnbp	−3.104e-08	**−3.539e-01**	−6.857e-02
Cyb5r3	3.785e-08	**8.065e-01**	5.937e-01
Dars2	−1.853e-07	3.554e-04	**4.985e-02**
Eef1a1	3.348e-08	−1.443e-04	**5.105e-01**
Hist1h2bf	−7.915e-08	6.322e-02	**1.346e-01**
Ptrh2	−3.072e-09	**−5.625e-01**	−6.945e-02
Zfp143	4.503e-09	−1.123e-02	**6.652e-02**

*Model of expression pattern for gene Eef1a1 is Model 3 in Table 3. The bold text represents the largest absolute values of weight in the regression coefficients of each gene*.

**Table 4:** Parameter values of model for gene Eef1a1.

**Gene name**	**Model**	**Regression coefficient**		
		**β_1_**	**β_2_**	**β_3_**
Eef1a1	2	2.510e-08	−2.282e-03	**5.448e-01**
Eef1a1	3	3.348e-08	−1.443e-04	**5.105e-01**

*The thickened data represent the largest absolute values of weight in regression coefficients of Model 2 and Model 3, respectively*.

Table 3 showed that regression coefficients β_2_ and β_3_ were large, which indicated that both distance and width had important influences on gene expression. Furthermore, distance had an effect on gene expression in the form of exponential function of time, and width had an effect on gene expression in the form of logarithmic function of time without other factors. Additionally, Table 4 shows that the difference of the regression coefficients between Model 2 and Model 3 were small. In both Model 2 and Model 3, β_3_ has the largest absolute values in regression coefficients for gene Eef1a1, which indicated that width was a key factor affecting gene expression.

### Performance Analysis of Our Proposed Model

Goodness of fit for proposed model was calculated to evaluate the performance of these models. In addition, performance of the models was tested by *F*-test and *t*-test. Results of goodness of fit, *F*-test and *t*-test are shown in Table 5. Results of goodness of fit, *F*-test and *t*-test of gene Eef1a1 are shown in Table 6.

**Table 5:** Goodness of fit, *F*-test and *t*-test for eight genes.

**Gene name**	**${\bar{R}}^{2}$**	***F* test**	***T*****-test**		
			***p*_1_**	***p*_2_**	***p*_3_**
Btbd8	0.9774	0.0003106	9.65e-05	0.019382	0.093871
Cnbp	0.9917	4.244e-05	4.75e-05	2.60e-05	0.0595
Cyb5r3	0.8929	0.006875	0.00590	0.00453	0.06944
Dars2	0.7298	0.04233	0.0771	0.0875	0.0154
Eef1a1	0.958	0.00107	0.016934	0.004609	0.000275
Hist1h2bf	0.8448	0.01431	0.00838	0.00518	0.00988
Ptrh2	0.8856	0.007835	0.0128	0.0105	0.0238
Zfp143	0.9046	0.005466	0.008996	0.077009	0.027986

*p_1_, p_2_ and p_3_ are p-values of t-test. Results of gene Eef1a1 in Table 5 are calculated by Model 3*.

**Table 6:** Goodness of fit, *F*-test and *t*-test for gene Eef1a1.

**Gene name**	**Model**		***F* test**	***T*-test**	
				***p*_1_**	***p*_2_**	***p*_3_**
Eef1a1	2	0.92	0.003858	0.07615	0.01737	0.00125
Eef1a1	3	0.958	0.00107	0.016934	0.004609	0.000275

*p_1_, p_2_, and p_3_ are p-values of t-test*.

Table 5 demonstrates that goodness of fit reached at least 80% for all genes except Dars2 by using our proposed method. In addition, the *p*-value of *F-*test and *t*-test were < 0.1, which meant that our proposed model was effective with 90% confidence.

As shown in Table 6, ${\bar{R}}^{2}$ from Model 3 was larger than Model 2, which means that distance had a greater influence on gene expression than time for gene Eef1a1.

As shown in Tables 3–6, absolute values of regression coefficients β_2_and β_3_were large in all regression coefficients. Additionally, the absolute value of regression coefficients for β_3_ was the largest in all regression coefficients with Model 2 and Model 3 for gene Eef1a1. Therefore, width was considered to be the most important factor affecting gene expression, and width had an effect on gene expression in the form of a logarithmic function.

### Analysis of Factors Affecting Gene Expression in Whole-Cell Developmental Stage

**Figure 3:**
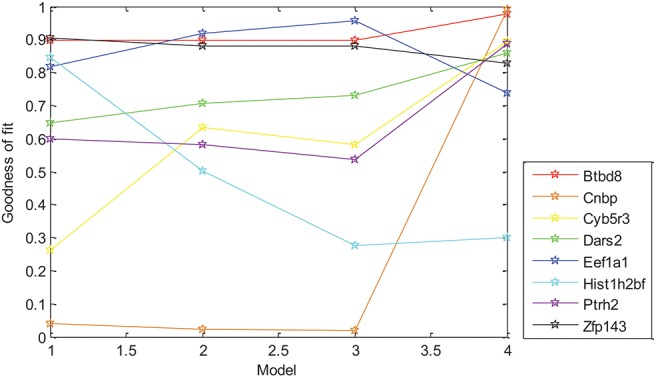
Effect of different models on gene expression patterns. Models 1–4 are represented from 1 to 4 on the x-axis, respectively. The y-axis represents corresponding goodness of fit.

In this paper, the relationship between gene expression and Oct4 combination intensity in promoter regions at the whole-cell developmental stage was analyzed based on the generalized linear regression model. Experimental results showed that the proposed model was effective for gene expression pattern of all eight selected genes except for Eef1a1. For exploring the effects of each model on the different genes, expression data of selected eight genes and Oct4 combination intensity in promoter regions were substituted into the models. Experimental results are shown in Figure 3.

Figure 3 demonstrated that differences in goodness of fit between different models for the same gene were large, which indicated that distance had strong effects on the gene expression of different genes with different levels. Strong correlation between gene expression and *D* × 0.5^*t*^, *W* × log_10_(*t* + 1) was found in Table 3, which indicated that distance had an effect on gene expression in the form of an exponential function of time, and width had an effect on gene expression in the form of a logarithmic function of time without other factors. However, goodness of fit from *D* × 0.5^*t*^ and *W* × log_10_(*t* + 1) was lower than for the selected six derived combination variables, which indicated that gene expression was promoted by the interaction of height, distance, width, and time.

### Analysis of Factors Affecting Gene Expression at Every Stage of Cell Development

**Figure 4:**
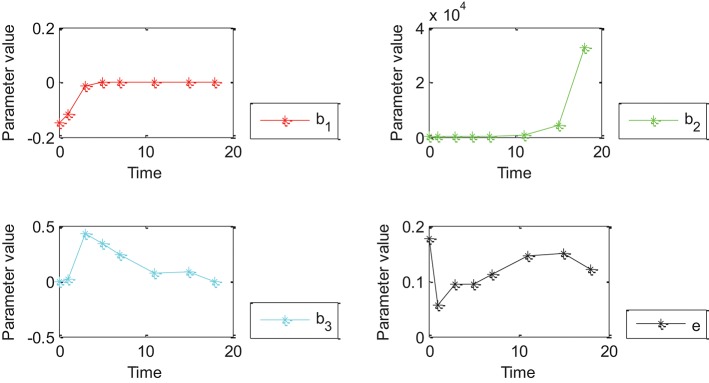
Effects of Oct4 combination intensity and time on gene expression at different stages of cell development for 27 genes. X-axis represents different stages of cell development after 0, 1, 3, 5, 7, 11, 15, and 18 day(s). Y-axis represents parameter value. Curves in red, green, blue, and black represent values of parameters β_1_, β_2_, β_3_ and ε from the proposed generalized linear regression model, respectively.

**Figure 5:**
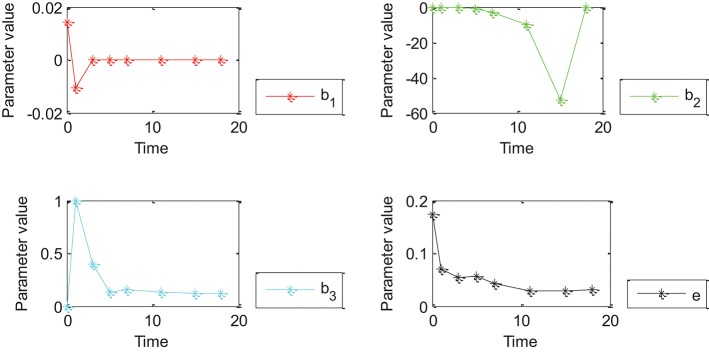
Effects of Oct4 combination intensity and time on gene expression at different stages of cell development for all genes from GEO. X-axis represents different stages of cell development after 0, 1, 3, 5, 7, 11, 15, and 18 days. Y axis represents parameter value. Curve in red, green, blue, and black represents values of parameter β_1_, β_2_, β_3_ and ε of the proposed generalized linear regression model, respectively.

Oct4 combination intensity and time had different effects on gene expression in different cell development stages. The goodness of fit obtained by Model 4 was higher than that obtained by Models 1–3 in the prediction of gene expression. Therefore, differences were analyzed based on Model 4 with testing data including 27 genes and all genes. Experimental results are shown in Figures 4, 5.

Figures 4, 5 show that the absolute value of β_3_ was larger than that of β_1_, β_2_, and e. Absolute values of β_1_and β_2_ were close to zero except for a few points, which indicated that width influenced gene expression in the form of a logarithmic function of time. However, change trends of β_1_ and β_2_ were different for Figures 4, 5. More specifically, the absolute value of β_1_ obtained by 27 genes decreased with time, and the value of was negative when time was equal to 0; the absolute value of obtained by all genes decreased with time and the value of was positive when time was equal to 0; the value of obtained by 27 genes was positive while value of obtained by all genes was negative due to partially missing data, which was contradictory and indicated that time had an important impact on gene expression. Incorrect conclusions were obtained when data of some certain time were missing. Therefore, Figures 4, 5 showed that width and time had important effects on gene expression. Furthermore, width influenced gene expression in the form of a logarithmic function of time.

### Application of Our Proposed Model in Gene Classification

Gene classification experiments were provided to test the generalization ability of the proposed model. Firstly, in order to avoid the influence of random disturbance on experimental results, the data of 27 genes, including height, distance, width and gene expression, were normalized. Then, Models 1–4 were applied to predict gene expression for 27 genes. Finally, the 27 genes were divided into two categories by comparing gene expression with a threshold; meanwhile, 10-fold cross-validation was used to test the model's performance. Comparison results of Models 1–4 showed Model 4 had a high goodness of fit. Therefore, 27 genes were classified by Model 4.

Gene groups of high and low expression were defined in an artificial way; meanwhile, threshold setting was random in the classification process. A BP neural network was used to classify positive and negative samples in order to prove that the randomness had little effect on experimental results. In this paper, the hidden layer of the BP neural network was set to one layer, and the number of hidden layer neurons was set to 2. In 10-fold cross-validation, regression coefficients and random disturbance of Model 4 were shown in Table 7. The prediction performance obtained by Model 4 and the BP neural network are shown in Table 8.

**Table 7:** Parameter values of Model 4 in 10-fold cross-validation.

**Serial** **number**	**Regression coefficient**			**Random** **disturbance**
	**β_1_**	**β_2_**	**β_3_**	**ε**
1	−0.19040	0.36415	1.94849	0.09097
2	−0.14777	0.34997	1.62791	0.08391
3	−0.19283	0.36895	1.34126	0.11151
4	−0.22430	0.32751	1.30305	0.12252
5	−0.17221	0.33449	1.45765	0.11442
6	−0.20314	0.35796	1.38494	0.11671
7	−0.15862	0.29230	1.26788	0.11184
8	−0.18597	0.36363	1.39271	0.10367
9	−0.20482	0.36602	1.37014	0.11242
10	−0.18064	0.34205	1.15955	0.11336

*1–10 represents the serial number of 10-fold cross-validation*.

**Table 8:** Prediction performance of different methods using 10-fold cross-validation.

**Methods**	**Performance evaluation standard**			
	**Acc**	***S*_*n*_**	***S*_*p*_**	**Mcc**
Model 4	**0.7643**	**0.8126**	**0.6947**	**0.5111**
BP neural network	0.7238	0.7585	0.6923	0.4537

*Bold text represents the maximum value of every performance evaluation criterion*.

Table 8 showed that the Acc, Sn, Sp, and Mcc obtained by Model 4 were the largest of the two different methods. Therefore, randomness of the threshold setting had little effect on experimental results, and our proposed method was effective in predicting gene expression.

## Conclusion

Cell reprogramming has been a hot issue in the field of life sciences and has played a significant role in medicine, such as in tissue repair, organ reconstruction, disease pathogenesis, and new drug development. Oct4 has especially played an important regulatory role in the process of cell reprogramming. However, there was no scientific method to quantify the relationship between Oct4 combination intensity and gene expression. Therefore, data from the eight selected typical genes were extracted from mouse transcriptome data and ChIP-seq data for quantifying the relationship between gene expression values and Oct4 combination intensity in promoter regions.

Firstly, a generalized linear regression model was constructed based on gene expression with eight different time periods during cell development and Oct4 combination intensity in promoter regions. Then, the relationship between Oct4 combination intensity and gene expression at whole and each stage of cell development was analyzed. Finally, the 27 genes were divided into positive and negative samples based on Model 4 and the BP neural network. Experimental results showed that width of combination influenced gene expression by a logarithmic function of time (day). Additionally, accuracy obtained by the models was 4.05% higher than that obtained by the BP neural network, which indicated that our proposed model was effective in predicting gene expression.

Several additional factors, including extent of histone modification, degree of chromatin opening, strength of promoter and binding sites of transcription factors and promoter regions, also affected gene expression. Non-linear relations between gene expression and Oct4 combination intensity were also ignored due to large non-linear relations. Therefore, in the future, multiple factors and non-linear relations should be considered to analyze key factors affecting gene expression.

## Author Contributions

SL: design experiment and analyze experiment result; ML: data processing and accomplish experiment; HL: extract and clean data from biological experiment and public database; YZ: provide idea from biological significance.

### Conflict of Interest Statement

The authors declare that the research was conducted in the absence of any commercial or financial relationships that could be construed as a potential conflict of interest.
